# Calprotectin Levels in HIV‐Infected Patients: Correlations With Disease Progression and Intestinal Inflammation

**DOI:** 10.1155/arat/5543700

**Published:** 2026-06-26

**Authors:** Huiyun Fan, Zhongyun Deng, Yong Qing, Zhen Rang, Fan Cui

**Affiliations:** ^1^ Department of Dermatology, People’s Hospital of Leshan, Leshan, China, leshan-hospital.com; ^2^ Department of Dermatology, Sichuan Provincial People’s Hospital Medical Group Chuantou Xichang Hospital, Xichang, China; ^3^ Department of Proctology and Dermatology, Chengdu Anorectal Hospital, Chengdu, China; ^4^ Institute of Dermatology, Sichuan Academy of Medical Sciences and Sichuan Provincial People’s Hospital, School of Medicine, University of Electronic Science and Technology of China, Chengdu, China, uestc.edu.cn

**Keywords:** calprotectin, HIV infection, intestinal mucosa, S100A8/A9

## Abstract

**Background:**

Persistent intestinal inflammation remains a significant concern in HIV‐infected patients despite effective antiretroviral therapy (ART). Calprotectin (S100A8/A9), a calcium‐binding protein complex involved in inflammatory signaling, has emerged as a potential biomarker in various inflammatory disorders; however, its role in HIV‐related intestinal inflammation has not been extensively studied.

**Methods:**

This study recruited 15 AIDS‐stage patients, 15 asymptomatic HIV‐infected patients, and 10 healthy controls from Chengdu Anal and Intestinal Specialized Hospital between April and August 2023. Serum, fecal samples, and colonic mucosal biopsies were collected. Calprotectin expression was quantified using enzyme‐linked immunosorbent assay (ELISA), immunohistochemistry, and Western blot analysis. Histopathological evaluation was performed using hematoxylin and eosin staining. Statistical correlations between calprotectin levels and clinical indicators, including CD4+ T‐cell counts, were analyzed.

**Results:**

Serum and fecal calprotectin concentrations were significantly elevated in HIV‐infected groups compared with controls, with the highest levels observed in AIDS‐stage patients (*p* < 0.05). Colonic mucosal inflammation severity correlated positively with S100A8/A9 protein expression (*p* < 0.05). Calprotectin expression inversely correlated with CD4+ T‐cell counts (serum: *r* = −0.509; feces: *r* = −0.520, both *p* < 0.001). Strong correlations were observed between calprotectin expression in serum, feces, and intestinal mucosal tissues.

**Conclusions:**

Calprotectin serves as a sensitive biomarker reflecting intestinal inflammation severity and immune dysfunction in HIV‐infected individuals. Serum and fecal calprotectin assays offer noninvasive, reliable methods for evaluating disease progression and inflammatory status and potentially guiding clinical management in HIV.

## 1. Background

Despite the significant benefits of antiretroviral therapy (ART), including effective viral suppression and restoration of circulating CD4+ T‐cell counts, intestinal mucosal barrier dysfunction and associated immune disturbances persist in HIV‐infected individuals [1]. Such disruptions typically induce breaches in the intestinal immune barrier, consequently initiating localized inflammatory responses. This inflammatory milieu promotes the infiltration and accumulation of neutrophils within the intestinal mucosa, some of which subsequently migrate into the intestinal lumen [2]. Activation of neutrophils and macrophages in this context facilitates the release and elevates the expression of calprotectin.

Calprotectin (S100A8/S100A9), a calcium‐ and zinc‐binding heterodimeric protein with an approximate molecular weight of 36 kDa, consists of S100A8 and S100A9 subunits linked by covalent bonds [3]. Representing nearly 60% of neutrophil cytosolic proteins, calprotectin is detected across diverse biological fluids, such as serum, saliva, cerebrospinal fluid, urine, and feces [4]. Exhibiting notable stability in calcium‐rich environments, calprotectin is an important arachidonic acid–binding protein within human neutrophils and participates critically in cytoskeletal rearrangement, leukocyte chemotaxis, arachidonic acid transport to inflammation sites, and orchestration of immune responses [5].

Calprotectin has emerged as an important biomarker for evaluating inflammatory conditions both within and beyond the gastrointestinal (GI) tract. Although considerable research has emphasized inflammatory bowel disease (IBD) [4, 6–8], calprotectin has also been investigated in psoriasis [5, 9, 10], systemic lupus erythematosus [11], rheumatoid arthritis [12], cardiovascular diseases [13, 14], and cancers such as oral squamous carcinoma [15], breast cancer [16], lung cancer [17, 18], and colorectal cancer [19]. In IBD specifically, fecal calprotectin (FCP) serves as a clinically recognized sensitive, specific, and noninvasive biomarker reflecting the presence and intensity of GI inflammation [7]. Additionally, elevated serum calprotectin concentrations, which typically decline following therapeutic intervention, correlate strongly with disease activity indices in rheumatoid arthritis, outperforming traditional markers such as C‐reactive protein (CRP) and erythrocyte sedimentation rate (ESR) [20]. Similar associations have also been reported in psoriatic arthritis [5].

Despite its well‐established clinical relevance in various inflammatory disorders, the role and diagnostic potential of calprotectin in HIV‐related GI inflammation remain inadequately investigated. Therefore, this study aims to bridge this critical knowledge gap by exploring the expression profiles of calprotectin (S100A8/A9) in blood, feces, and intestinal tissues, evaluating its potential as a reliable, noninvasive biomarker for intestinal mucosal inflammation in HIV‐infected patients.

## 2. Materials and Methods

### 2.1. Subjects

Participants for this study were recruited from Chengdu Anal and Intestinal Specialized Hospital, adhering strictly to the “Diagnosis of AIDS and HIV Infection” guidelines provided by the National Health Commission of the People’s Republic of China [21]. The study included 15 patients diagnosed with AIDS (Group A), 15 asymptomatic HIV‐infected patients (Group B), and 10 healthy controls who had undergone routine colonoscopic examinations at the hospital (Group C). Recruitment and data collection occurred from April 2023 to August 2023. Ethical approval was obtained from the Ethics Committee of Chengdu Anal and Intestinal Specialized Hospital (Approval No. 105, 2023), and all subjects provided informed consent before participation.

### 2.2. Inclusion Criteria

#### 2.2.1. Group A

1. Confirmed HIV infection characterized by CD4+ T‐cell counts of less than 200 cells/μL or diagnosis of at least one AIDS‐defining illness according to established criteria.
2. Age ranging from 18 to 60 years.
3. No history of cardiovascular, endocrine, digestive, and connective tissue disorders (such as rheumatoid arthritis or systemic lupus erythematosus), dermatological conditions (e.g., psoriasis), or malignancies.
4. No episodes of acute infections or persistent GI symptoms such as abdominal pain or diarrhea within the preceding month.
5. No intake of nonsteroidal anti‐inflammatory drugs (NSAIDs), proton‐pump inhibitors (PPIs), or related medications within the month preceding recruitment.

#### 2.2.2. Group B

1. HIV‐positive status confirmed via antibody testing with CD4+ T‐cell counts exceeding 200 cells/μL.
2. All other inclusion criteria identical to those outlined for Group A.

#### 2.2.3. Group C (Healthy Controls)

1. HIV‐negative status confirmed via antibody testing and presenting normal CD4+ T‐cell counts.
2. All other inclusion criteria identical to those outlined for Group A.

### 2.3. Sample Collection

#### 2.3.1. Blood and Fecal Samples

Blood samples were collected from all participants after an overnight fast. Samples were drawn in the morning, centrifuged immediately to obtain plasma, aliquoted, and then stored at −80 °C for subsequent analyses. Fecal samples were self‐collected by participants at home using sterile containers, promptly refrigerated, and delivered to the laboratory within 2 h. Samples were stored at −80 °C until further analysis.

#### 2.3.2. Intestinal Mucosal Samples

From each participant, two biopsy specimens (approximately 0.5 cm × 0.5 cm × 0.2 cm each) were obtained via colonoscopy from lesion‐free mucosal regions of the distal descending colon. Samples were processed immediately for hematoxylin and eosin (HE) staining, immunohistochemical staining, and Western blot analysis to evaluate intestinal mucosal expression levels of S100A8 protein.

### 2.4. Enzyme‐Linked Immunosorbent Assay (ELISA)

Collected blood and fecal samples were centrifuged to obtain clear supernatants for analysis. Calprotectin concentrations were quantified using commercial ELISA kits following the manufacturer’s instructions. Briefly, standards and sample supernatants were added to ELISA plates, followed by incubation with enzyme‐linked conjugate. After rigorous washing, substrate solution was added, and reactions were terminated using a stop solution. Optical density (OD) values were recorded at a wavelength of 450 nm. Calprotectin concentrations in samples were calculated based on standard curves generated from known concentrations provided in the kit.

### 2.5. HE Staining

Colonic tissue samples were fixed in formalin, embedded in paraffin, sectioned, and subsequently stained using standard HE staining protocols. The stained tissue sections were examined microscopically by experienced pathologists to evaluate histological characteristics, including the degree of inflammation and infiltration of neutrophils within the colonic mucosa.

### 2.6. Immunohistochemistry

Paraffin‐embedded sections were deparaffinized, rehydrated, and subjected to antigen retrieval. After blocking nonspecific binding, sections were incubated with primary antibodies specific to S100A8 and S100A9, followed by secondary antibodies. Diaminobenzidine (DAB) was used for color development, and nuclei were counterstained. Sections were then dehydrated and sealed. Initial observation was performed at low magnification, followed by high magnification to analyze areas showing positive staining of S100A8 and S100A9.

### 2.7. Western Blotting

Intestinal mucosa tissues were lysed, and the supernatant was collected post‐centrifugation. Proteins were denatured and subjected to electrophoresis, followed by transfer to a membrane. After blocking, the membrane was incubated with primary and secondary antibodies. Chemiluminescence was conducted in a darkroom to visualize the proteins. Images were then processed for analysis, and grayscale values were quantified.

### 2.8. Statistical Analysis

Statistical analyses were performed using SPSS software (Version 26.0, IBM Corp., Armonk, NY, USA). Normality of data distribution was assessed using the Shapiro–Wilk test. Comparisons among multiple groups were conducted using one‐way ANOVA or the Kruskal–Wallis test, depending on data distribution, with post hoc analyses using the least significant difference (LSD) test for parametric data or Mann–Whitney *U* test for nonparametric data. Correlation analyses were conducted using Spearman’s rank correlation coefficient. Data are presented as mean ± standard deviation (SD). A *p* value less than 0.05 was considered statistically significant.

## 3. Results

### 3.1. Clinical and Demographic Characteristics

Clinical and demographic characteristics of all participants are summarized in Table 1. All participants enrolled were male. Age was normally distributed and compared across groups using one‐way ANOVA. CD4+ T‐cell counts and ART durations were not normally distributed and thus analyzed using the Mann–Whitney *U* test. No significant differences were observed between Groups A and B concerning age (*p* = 0.301) or duration of ART (*p* = 0.076). However, CD4+ T‐cell counts differed significantly among groups: Group A had significantly lower CD4+ counts compared to Group B (*p* < 0.001) and Group C (*p* < 0.001), and Group B had lower counts than Group C (*p* = 0.048).

**TABLE 1:** Clinical and demographic characteristics of participants.

	Group A (*n* = 15)	Group B (*n* = 15)	Group C (*n* = 10)	*F*	*p*	Group A vs Group B	Group A vs Group C	Group B vs Group C
Age, years,	38.87 ± 10.99	35.93 ± 9.18	42.90 ± 12.81	1.24	0.301	*p* = 0.463	*p* = 0.368	*p* = 0.124
CD4+ T‐cell count, cells/μL	128 (94, 322)	489 (393, 823)	712 (616,887)	/	< 0.001	*p* < 0.001	*p* < 0.001	*p* = 0.048
Duration of ART, years	15 (1, 84)	60 (36, 84)	/	/	0.076	*p* = 0.076	/	/

### 3.2. ELISA for Calprotectin Levels

Serum calprotectin and FCP levels across the three groups did not exhibit normal distributions and thus were analyzed using the Mann–Whitney *U* test. Serum calprotectin levels were significantly elevated in both Group A and Group B compared to Group C (*p* < 0.001, *p* = 0.036, respectively), with Group A having higher levels than Group B (*p* = 0.016). FCP levels were also significantly higher in Groups A and B compared to Group C (*p* = 0.002, *p* = 0.031, respectively); however, no significant difference was noted between Groups A and B (*p* = 0.367). These results are detailed in Table 2.

**TABLE 2:** Serum and fecal calprotectin results in the three study groups.

	Group A (*n* = 15)	Group B (*n* = 15)	Group C (*n* = 10)	*F*	*p*	Group A vs Group B	Group A vs Group C	Group B vs Group C
Serum calprotectin (ng/mL)	182.2 (144.6, 310.3)	133.8 (113.8, 179.2)	106.6 (81.4, 138.6)	/	0.001	*p* = 0.016	*p* < 0.001	*p* = 0.036
Fecal calprotectin (ng/mL)	94.2 (65.5, 137.1)	67.7 (58.3, 138.1)	48.3 (34.9, 71.1)	/	0.001	*p* = 0.367	*p* = 0.002	*p* = 0.031

### 3.3. Histopathological Assessment (HE Staining)

**FIGURE 1:**
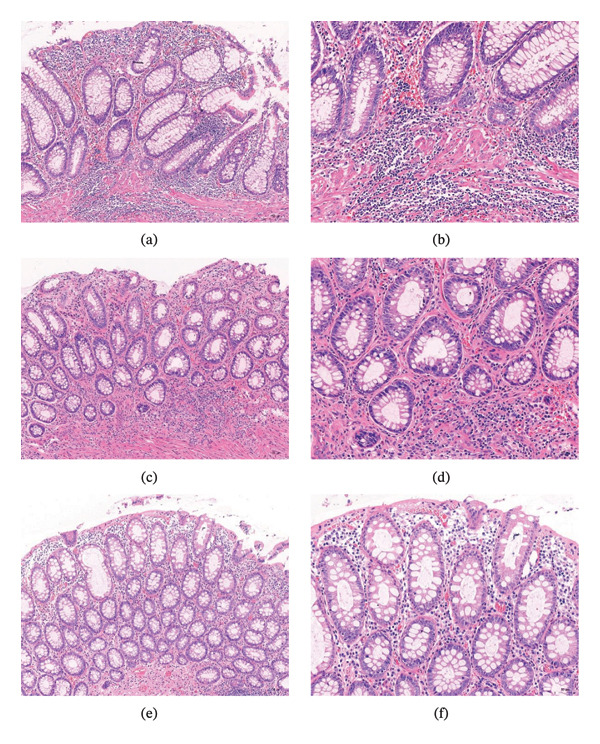
HE staining results of the three study groups. (a) Group A (100×); (b) Group A (200×); (c) Group B (100×); (d) Group B (200×); (e) Group C (100×); (f) Group C (200×).

Histological evaluation revealed no significant inflammatory infiltration in colonic tissues from healthy controls (Group C). In contrast, marked infiltration of inflammatory cells and extensive lamina propria necrosis were observed in Groups A and B. Necrotic areas were characterized by loss or reduction of intestinal glands, with visible necrotic cellular debris exhibiting pyknotic and lysed nuclei. These pathological features were notably more severe in Group A. Representative histological findings are presented in Figure 1.

### 3.4. Neutrophil Counts in Colonic Mucosa

Neutrophil counts in intestinal tissues followed normal distribution and homogeneity of variance; thus, comparisons were made using one‐way ANOVA. Group A showed significantly elevated neutrophil counts compared to both Group C (*p* = 0.006) and Group B (*p* = 0.027). While Group A exhibited higher neutrophil counts compared to Group B, this difference did not reach statistical significance (*p* = 0.275). Data are summarized in Table 3.

**TABLE 3:** Results of neutrophil count in intestinal tissues.

	Group A (*n* = 15)	Group B (*n* = 15)	Group C (*n* = 10)	F/P	Group A vs Group B	Group A vs Group C	Group B vs Group C
Neutrophil count (cells/HPF)	20.45 ± 6.52	15.78 ± 3.56	4.44 ± 3.56	8.98/0.016	*p* = 0.275	*p* = 0.006	*p* = 0.027

### 3.5. Immunohistochemical Analysis of S100A8 and S100A9

**FIGURE 2:**
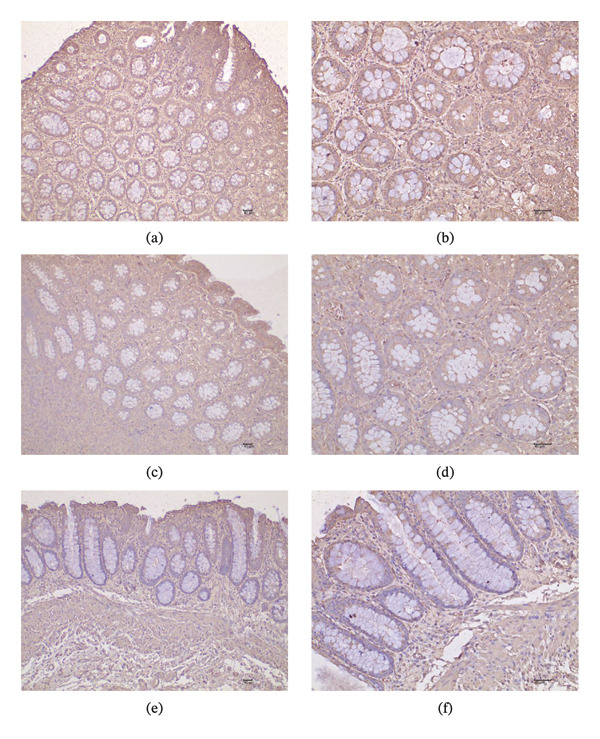
Immunohistochemical expression of S100A8 in study subjects across three Groups. (a) Group A (100×); (b) Group A (200×); (c) Group B (100×); (d) Group B (200×); (e) Group C (100×); (f) Group C (200×).

**FIGURE 3:**
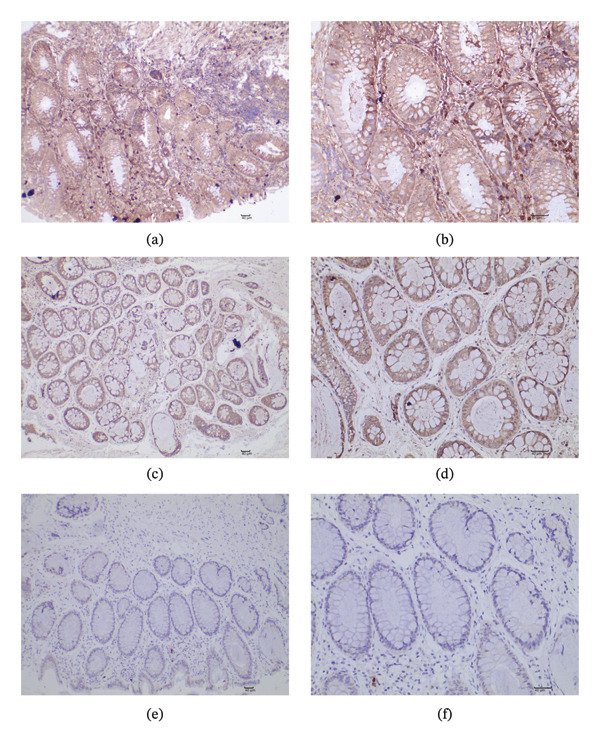
Immunohistochemical expression of S100A9 in study subjects across three groups. (a) Group A (100×); (b) Group A (200×); (c) Group B (100×); (d) Group B (200×); (e) Group C (100×); (f) Group C (200×).

Immunohistochemistry revealed that S100A8 and S100A9 proteins were expressed primarily in the cytoplasm, with occasional nuclear localization (Figures 2 and 3). Statistical analysis using one‐way ANOVA confirmed significantly higher positivity rates for S100A8 in Group A compared to Groups B and C (*p* = 0.032 and *p* < 0.001, respectively), with Group B showing higher positivity than Group C (*p* = 0.014). Similarly, the positivity rate for S100A9 was significantly greater in Group A than Groups B and C (*p* = 0.023 and *p* < 0.001, respectively) and higher in Group B compared to Group C (*p* = 0.001). These findings are summarized in Table 4.

**TABLE 4:** Immunohistochemical expression of S100A8 and S100A9.

	Group A (*n* = 15)	Group B (*n* = 15)	Group C (*n* = 10)	F/P	Group A vs Group B	Group A vs Group C	Group B vs Group C
The percentage of S100A8‐positive	24.130 ± 2.845	18.505 ± 4.863	11.837 ± 2.914	14.120/0.001	*p* = 0.032	*p* < 0.001	*p* = 0.014
The percentage of S100A9‐positive	20.119 ± 5.728	13.669 ± 3.269	2.208 ± 1.487	27.004/< 0.001	*p* = 0.023	*p* < 0.001	*p* = 0.001

### 3.6. Western Blot Analysis of S100A8 and S100A9 Expression

**FIGURE 4:**
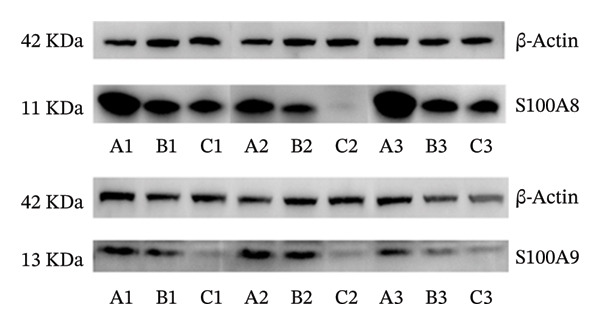
Western blotting bands of S100A8 and S100A9 in intestinal tissues. A1, A2, and A3 are AIDS groups (Group A); B1, B2, and B3 are asymptomatic HIV‐infected groups (Group B); C1, C2, and C3 are healthy controls (Group C).

Western blot analysis from representative samples is depicted in Figure 4. After normalization to the internal control *β*‐actin, grayscale intensity values of S100A8 and S100A9 conformed to normal distribution and homogeneity of variance, allowing analysis using one‐way ANOVA. Expression levels of S100A8 and S100A9 were significantly elevated in both Groups A (*p* = 0.002, *p* = 0.003) and B (*p* = 0.035, *p* = 0.034) relative to Group C. Although Group A displayed higher expression of both proteins compared to Group B, these differences were not statistically significant (*p* = 0.053, *p* = 0.210, respectively). These results are summarized in Table 5.

**TABLE 5:** Grayscale values of S100A8 and S100A9 in Western blotting.

	Group A (*n* = 15)	Group B (*n* = 15)	Group C (*n* = 10)	F/P	Group A vs Group B	Group A vs Group C	Group B vs Group C
S100A8 grayscale values	3.246 ± 0.622	2.191 ± 0.395	1.000 ± 0.571	13.078/0.006	*p* = 0.053	*p* = 0.002	*p* = 0.035
S100A9 grayscale values	4.188 ± 1.720	3.050 ± 1.600	1.000 ± 0.159	7.075/0.009	*p* = 0.210	*p* = 0.003	*p* = 0.034

### 3.7. Correlation Analysis

Correlation analyses were performed using Pearson’s correlation for normally distributed data and Spearman’s correlation for nonnormally distributed data.

**FIGURE 5:**
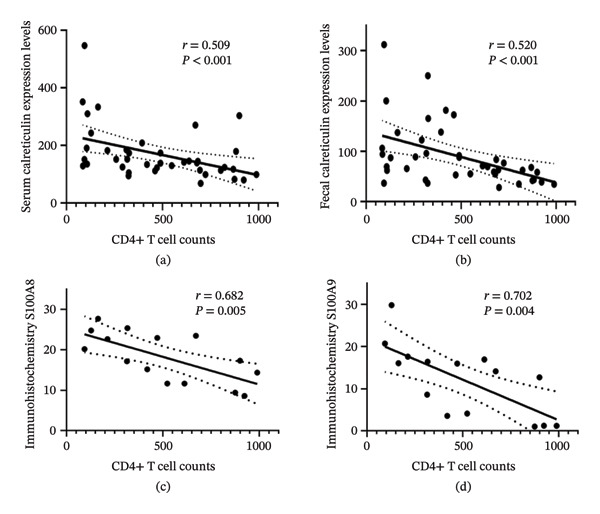
Correlation of cellular immunity levels with calprotectin expression levels. (a) Negative correlation between CD4+ T‐cell counts and serum calprotectin expression levels. (b) Negative correlation between CD4+ T‐cell counts and fecal calprotectin expression levels. (c) Negative correlation between CD4+ T‐cell counts and S100A8 expression in immunohistochemistry. (d) Negative correlation between CD4+ T‐cell counts and S100A9 expression in immunohistochemistry.

**FIGURE 6:**
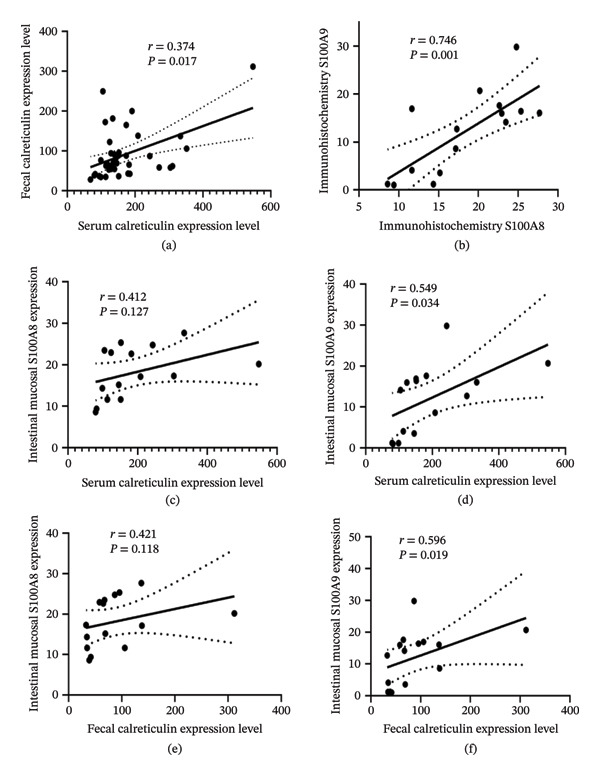
Correlation of calprotectin expression levels among tissues. (a) Positive correlation between serum and fecal calprotectin expression levels. (b) Positive correlation between S100A8 and S100A9 expression in the intestinal mucosa. (c) Positive correlation between serum calprotectin and S100A8 expression in the intestinal mucosa. (d) Positive correlation between serum calprotectin and S100A9 expression in the intestinal mucosa. (e) Positive correlation between fecal calprotectin and S100A8 expression in the intestinal mucosa. (f) Positive correlation between fecal calprotectin and S100A9 expression in the intestinal mucosa.

1. CD4+ T‐cell counts and calprotectin expression: CD4+ T‐cell counts exhibited significant negative correlations with calprotectin levels in serum (*r* = −0.509, *p* < 0.001) and feces (*r* = −0.520, *p* < 0.001) (Figure 5).
2. CD4+ T‐cell counts and intestinal mucosal S100A8/S100A9 expression: Significant negative correlations were also observed between CD4+ T‐cell counts and intestinal mucosal expression of S100A8 (*r* = −0.682, *p* = 0.005) and S100A9 (*r* = −0.702, *p* = 0.004) (Figure 5).
3. Correlations between calprotectin expressions across different tissues:
  1. Serum calprotectin and FCP levels showed a positive correlation (*r* = 0.374, *p* = 0.017).
  2. Strong positive correlations were observed between intestinal mucosal expressions of S100A8 and S100A9 (*r* = 0.746, *p* = 0.001).
  3. Serum calprotectin levels correlated positively with intestinal mucosal S100A8 (*r* = 0.412, *p* = 0.127) and significantly with S100A9 (*r* = 0.549, *p* = 0.034).
  4. FCP showed positive correlations with intestinal mucosal S100A8 (*r* = 0.421, *p* = 0.118) and significant correlations with S100A9 (*r* = 0.596, *p* = 0.019) (Figure 6).

## 4. Discussion

Calprotectin, specifically the S100A8/A9 complex, belongs to the S100 protein family and exerts multiple biological functions, including roles in antimicrobial responses, regulation of cellular proliferation, and apoptosis induction [5, 22]. Our findings demonstrate that HIV infection activates an inflammatory cascade within the intestinal mucosa, recruiting neutrophils and other immune cells. These cells release calprotectin into the intestinal lumen, directly contributing to mucosal inflammation and subsequent tissue damage.

In this study, we observed significantly elevated serum calprotectin levels in HIV‐infected patients compared with healthy controls. Notably, the highest calprotectin concentrations were detected in AIDS‐stage patients, correlating negatively with CD4+ T‐cell counts [23, 24]. This relationship aligns with previous research demonstrating that calprotectin correlates with inflammatory disease markers in conditions such as IBD, where FCP mirrors clinical disease activity and CRP levels [25]. FCP has been included in the management guidelines for adult and pediatric IBD by multiple major associations. It serves as a noninvasive tool to screen for IBD with high sensitivity and to predict clinical recurrence [6]. In symptomatic IBD patients, an FCP level < 250 μg/g helps distinguish inflammatory flares from noninflammatory conditions, while levels < 100 μg/g in patients in clinical remission can indicate mucosal healing and a lower risk of short‐term relapse [6]. Similar findings have been documented in rheumatoid arthritis, where serum calprotectin levels parallel disease progression and inflammatory activity [20, 26]. Our results reinforce calprotectin’s potential as a reliable biomarker for systemic inflammation and immune dysregulation, extending its relevance from established inflammatory disorders to the context of HIV infection.

The significant elevation of serum calprotectin levels observed in our study emphasizes its diagnostic potential for assessing systemic inflammation. Furthermore, the inverse correlation between calprotectin levels and CD4+ T‐cell counts in HIV‐infected patients provides unique insights into HIV pathophysiology, highlighting a direct link between systemic inflammation, immune impairment, and disease severity. This suggests that serum calprotectin could serve as a clinically relevant indicator to monitor disease progression and therapeutic responses in HIV infection.

Histopathological examination of intestinal biopsies revealed severe mucosal inflammation and tissue damage in HIV‐infected patients, particularly those in the AIDS stage. Immunohistochemical and Western blot analyses consistently demonstrated elevated mucosal expression of S100A8 and S100A9 proteins, which correlated positively with the severity of intestinal lesions. These proteins are integral to neutrophil activation and recruitment, suggesting their active involvement in sustaining inflammation and mucosal injury [22, 27–29]. Additionally, S100A8/A9 may influence HIV pathogenesis by modulating immune responses, potentially exacerbating local inflammation or affecting viral replication dynamics.

FCP levels were significantly higher in HIV‐infected subjects than in healthy controls, although no significant difference was found between symptomatic and asymptomatic patients. This observation is consistent with previous studies conducted in pediatric HIV‐infected populations [30–32], suggesting that FCP concentrations may be influenced by factors such as age, gut microbiota diversity, immune maturity, and other individual variables. Studies have shown that following HIV infection, levels of the intestinal immune marker zonulin decrease in the gut but increase in plasma [33]. This redistribution is associated with the loss of intestinal CD4+ T cells and increased intestinal inflammation, suggesting that elevated systemic zonulin levels correlate with disease progression and intestinal damage [33]. Furthermore, lipopolysaccharide (LPS) is a core marker of bacterial translocation. In HIV‐infected patients, plasma LPS levels correlate directly with various inflammatory markers in plasma and cerebrospinal fluid, as well as with the degree of blood–brain barrier permeability. Elevated plasma LPS, neuroinflammation, and blood–brain barrier dysfunction have been observed in untreated HIV‐infected patients. These findings indicate that the degree of microbial translocation is linked to neuroinflammation [34]. Such complexity underscores the importance of further investigations aimed at elucidating the precise dynamics governing intestinal inflammation in HIV‐infected populations.

This study has several limitations. First, the sample size was relatively small, limiting statistical power. Additionally, the exclusive recruitment of male participants from a single hospital restricts the generalizability of our findings. Geographic factors, socioeconomic status, and methodological constraints might also introduce biases. Future studies should involve larger, more diverse populations to confirm these preliminary findings and explore calprotectin’s role comprehensively across varying demographic and clinical contexts.

## 5. Conclusion

Calprotectin levels in serum and feces correlate significantly with intestinal inflammation severity and inversely correlate with cellular immune status in HIV‐infected individuals. Given their noninvasive nature and diagnostic potential, calprotectin assays in serum and feces could substantially enhance clinical management by serving as practical biomarkers for monitoring HIV‐associated inflammation, disease progression, and therapeutic effectiveness.

## Author Contributions

Fan Cui and Zhongyun Deng designed the research. Huiyun Fan and Zhongyun Deng accessed and verified the data, performed statistical analysis, and drafted the manuscript. All the authors participated in the interpretation of the results and critical revision of the manuscript.

## Funding

No funding was received for this research.

## Consent

The authors have nothing to report.

## Conflicts of Interest

The authors declare no conflicts of interest.

## Data Availability

Research data are not shared.

## References

[1] Ruiz-Briseno M. D. R. , De Arcos-Jimenez J. C. , Ratkovich-Gonzalez S. et al., Association of Intestinal and Systemic Inflammatory Biomarkers with Immune Reconstitution in HIV+ Patients on ART, Journal of Inflammation. (2020) 17, no. 1, 10.1186/s12950-020-00262-4. PMC7558748 33071649

[2] Ma S. , D J. E. , Claire D. et al., Gut Epithelial Barrier and Systemic Inflammation During Chronic HIV Infection, AIDS. (2015) 29, no. 1, 43–51. 25387317 10.1097/QAD.0000000000000511 PMC4444362

[3] Wang S. , Song R. , Wang Z. , Jing Z. , and Ma J. , S100A8/A9 in Inflammation, Frontiers in Immunology. (2018) 9, 10.3389/fimmu.2018.01298. PMC6004386 29942307

[4] Ayling R. M. , Kok K. F. , and Calprotectin , Fecal Calprotectin, Advances in Clinical Chemistry. (2018) 87, 161–190, 10.1016/bs.acc.2018.07.005. 30342711

[5] Donato R. , Cannon B. R. , Sorci G et al., Functions of S100 Proteins, Current Molecular Medicine. (2013) 13, no. 1, 24–57, 10.2174/156652413804486214. 22834835 PMC3707951

[6] Ricciuto A. and Griffiths A. M. , Clinical Value of Fecal Calprotectin, Critical Reviews in Clinical Laboratory Sciences. (2019) 56, no. 5, 307–320, 10.1080/10408363.2019.1619159. 31088326

[7] D′amico F. , Rubin D. T. , Kotze P. G. et al., International Consensus on Methodological Issues in Standardization of Fecal Calprotectin Measurement in Inflammatory Bowel Diseases, United European Gastroenterology Journal. (2021) 9, no. 4, 451–460, 10.1002/ueg2.12069. 33961734 PMC8259254

[8] Bertani L. , Mumolo M. G. , Tapete G. et al., Fecal Calprotectin: Current and Future Perspectives for Inflammatory Bowel Disease Treatment, European Journal of Gastroenterology and Hepatology. (2020) 32, no. 9, 1091–1098, 10.1097/meg.0000000000001731. 32282400

[9] Roseth A. G. , Fagerhol M. K. , Aadland E. , and Schjønsby H. , Assessment of the Neutrophil Dominating Protein Calprotectin in Feces-a Methodologic Study, Scandinavian Journal of Gastroenterology. (1992) 27, no. 9, 793–798, 10.3109/00365529209011186. 1411288

[10] Jukic A. , Bakiri L. , Wagner E. F. , Tilg H. , and Adolph T. E. , Calprotectin: from Biomarker to Biological Function, Gut. (2021) 70, no. 10, 1978–1988, 10.1136/gutjnl-2021-324855. 34145045 PMC8458070

[11] Homa-Mlak I. , Mazurek M. , Majdan A. , Mlak R. , and Mełecka-Massalska T. , Serum Calprotectin a Netproduct-as a Biomarker of Disease Activity in Patients with Systemic Lupus Erythematosus: a Single-Center Case-Control Study from Poland, Medical Science Monitor. (2022) 28, 10.12659/msm.936534. PMC9290428 35821629

[12] Jarlborg M. , Courvoisier D. S. , Lamacchia C. et al., Serum Calprotectin: a Promising Biomarker in Rheumatoid Arthritis and Axial Spondyloarthritis, Swiss Medical Weekly. (2020) . 10.1186/s13075-020-02190-3 PMC7201559 32375861

[13] Sreejit G. , Latif A. A. , Murphy A. J. , and Nagareddy P. R. , Emerging Roles of neutrophil-borne S100A8/A9 in Cardiovascular Inflammation, Pharmacological Research. (2020) 161, 10.1016/j.phrs.2020.105212. PMC7755830 32991974

[14] Schiopu A. and Cotoi O. S. , S100A8 and S100A9: Damps at the Crossroads Between Innate Immunity, Traditional Risk Factors, and Cardiovascular Disease, Mediators of Inflammation. (2013) 2013, 828354–10, 10.1155/2013/828354. 24453429 PMC3881579

[15] Argyris P. P. , Slama Z. , Malz C. et al., Intracellular Calprotectin (S100A8/A9)controls Epithelial Differentiation and caspase-mediated Cleavage of EGFR in Head and Neck Squamous Cell Carcinoma, Oral Oncology. (2019) 95, 1–10, 10.1016/j.oraloncology.2019.05.027. 31345374 PMC6662626

[16] Bao Y. , Wang A. , and Mo J. , S100A8/A9 is Associated with Estrogen Receptor Loss in Breast Cancer, Oncology Letters. (2016) 11, no. 3, 1936–1942, 10.3892/ol.2016.4134. 26998104 PMC4774479

[17] Sumardika I. W. , Chen Y. , Tomonobu N. et al., Neuroplastin-Mediates S100A8/A9-induced Lung Cancer Disseminative Progression, Molecular Carcinogenesis. (2019) 58, no. 6, 980–995, 10.1002/mc.22987. 30720226

[18] Cho S. B. , Kim I. K. , Yeo C. D. , and Lee S. H. , Association Between Clinicopathological Parameters and S100A8/A9 Expression According to Smoking History in Patients with Non-small Cell Lung Cancer, In Vivo. (2024) 38, no. 1, 474–481, 10.21873/invivo.13462. 38148054 PMC10756484

[19] Kim J. H. , Oh S.-H. , Kim E.-J. et al., The Role of Myofibroblasts in Upregulation of S100A8 and S100A9 and the Differentiation of Myeloid Cells in the Colorectal Cancer Microenvironment, Biochemical and Biophysical Research Communications. (2012) 423, no. 1, 60–66, 10.1016/j.bbrc.2012.05.081. 22634002

[20] José M. I. , Beatriz S. F. , and Raimon S. , From Bench to Bedside: Calprotectin (S100A8/S100A9) as a Biomarker in Rheumatoid Arthritis, Frontiers in Immunology. (2022) 13. 10.3389/fimmu.2022.1001025 PMC9672845 36405711

[21] Health Industry Standard of the People’s Republic of China , Diagnosis of AIDS and HIV Infection(Ws 293-2019)[S], 2019.

[22] yckman C. , Robichaud G. A. , Roy J. et al., HIV-1 Transcription and Virus Production are Both Accentuated by the Proinflammatory myeloid-related Proteins Inhuman CD4+ T Lymphocytes, Journal of Immunology. (2002) 169, no. 6, 3307–3313. 10.4049/jimmunol.169.6.3307 12218151

[23] Muller F. , Froland S. S. , Aukrust P. et al., Elevated Serum Calprotectin Levelsin HIV-Infected Patients: the Calprotectin Response During ZDV Treatment is Associated with Clinical Events, Journal of Acquired Immune Deficiency Syndromes & Human Retrovirology. (1994) 7, no. 9, 931–939. 7914232

[24] Strasser F. , Gowland P. L. , and Ruef C. , Elevated Serum Macrophage Inhibitory factor-related Protein (MRP) 8/14 Levels in Advanced HIV Infection and During Disease Exacerbation, Journal of Acquired Immune Deficiency Syndromes. (1997) 16, no. 4, 230–238, 10.1097/00042560-199712010-00002. 9402068

[25] Mori A. , Mitsuyama K. , Sakemi R. et al., Evaluation of Serum Calprotectin Levels in Patients with Inflammatory Bowel Disease, The Kurume Medical Journal. (2021) 66, no. 4, 209–215, 10.2739/kurumemedj.ms664009. 34690210

[26] Klingberg E , Carlsten H. , Hilme E. , Hedberg M. , and Forsblad-d’Elia H. , Calprotectin in Ankylosing spondylitis--Frequently Elevated in Feces, but Normal in Serum, Scandinavian Journal of Gastroenterology. (2012) 47, no. 4, 435–444, 10.3109/00365521.2011.648953. 22229862

[27] Endoh Y. , Chung Y. M. , Clark I. A. , Geczy C. L. , and Hsu K. , IL-10-dependent S100A8 Gene Induction in monocytes/macrophages by double-stranded RNA, Journal of immunology Baltimore. (2009) 182, no. 4, 2258–2268, 10.4049/jimmunol.0802683. 19201880

[28] Maarifi G. , Lagisquet J. , Hertel Q. et al., Alarmin S100A9 Restricts Retroviralinfection by Limiting Reverse Transcription in Human Dendritic Cells, EMBO Journal. (2021) 40, no. 16, 10.15252/embj.2020106540. PMC8365256 34121210

[29] Oguariri R. M. , Brann T. W. , Adelsberger J. W. et al., Short Communication: S100A8 and S100A9, Biomarkers of SARS-CoV-2 Infection and Other Diseases, Suppress HIV Replication in Primary Macrophages, AIDS Research and Human Retroviruses. (2022) 38, no. 5, 401–405, 10.1089/aid.2021.0193. 35045753 PMC9131038

[30] Mantegazza C. , Maconi G. , Giacomet V. et al., Gut and Mesenteric Lymph Node Involvement in Pediatric Patients Infected with Human Immunodeficiency Virus, HIV/AIDS (Auckland, NZ). (2014) 6, 69–74. 10.2147/HIV.S60157 PMC4020886 24855391

[31] Blaauw J. , Chikwana J. , Chaima D. et al., The Presence of Enteropathy in Hiv Infected Children on Antiretroviral Therapy in Malawi, PLoS One. (2024) 19, no. 2, 10.1371/journal.pone.0298310. PMC10852317 38330085

[32] Hestvik E. , Olafsdottir E. , Tylleskar T. et al., Faecal Calprotectin in hiv-infected, haart-naive Ugandan Children, Journal of Pediatric Gastroenterology and Nutrition. (2012) 54, no. 6, 785–790, 10.1097/mpg.0b013e318241a683. 22108340

[33] Augustin M. , Horn C. , Ercanoglu M. S. et al., From Gut to Blood: Redistribution of Zonulin in People Living with HIV, Biomedicines. (2024) 12, no. 10, 10.3390/biomedicines12102316. PMC11505231 39457626

[34] Jiang W. , Luo Z. , Stephenson S. et al., Cerebrospinal Fluid and Plasma Lipopolysaccharide Levels in Human Immunodeficiency Virus Type 1 Infection and Associations with Inflammation, Blood-Brain Barrier Permeability, and Neuronal Injury, The Journal of Infectious Diseases. (2021) 223, no. 9, 1612–1620, 10.1093/infdis/jiaa765. 33320240 PMC8136977

